# Tethered Lipid Membranes as a Nanoscale Arrangement towards Non-Invasive Analysis of Acute Pancreatitis

**DOI:** 10.3390/biomedicines9070755

**Published:** 2021-06-29

**Authors:** Rima Budvytyte, Akvile Milasiute, Dalius Vitkus, Kestutis Strupas, Aiste Gulla, Ieva Sakinyte, Julija Razumiene

**Affiliations:** 1 Life Sciences Center, Institute of Biochemistry, Vilnius University, Sauletekio av. 7, LT-10257 Vilnius, Lithuania; akvile.milasiute@gmc.stud.vu.lt (A.M.); ieva.sakinyte@gmc.vu.lt (I.S.); 2 Institute of Biomedical Sciences, Faculty of Medicine, Vilnius University, M.K.Ciurlionio St. 21, LT-03101 Vilnius, Lithuania; dalius.vitkus@santa.lt; 3 Institute of Clinical Medicine, Faculty of Medicine, Vilnius University, M.K.Ciurlionio St. 21, LT-03101 Vilnius, Lithuania; kestutis.strupas@santa.lt (K.S.); aiste.kielaite-gulla@santa.lt (A.G.)

**Keywords:** HSP70 and HSP90, severity of acute pancreatitis, tethered bilayer lipid membranes, electrochemical impedance spectroscopy, urine

## Abstract

Extracellular heat shock proteins (HSPs) mediate immunological functions and are involved in pathologies such as infection, stress, and cancer. Here, we demonstrated the dependence of an amount of HSP70 and HSP90 in serum vs. severity of acute pancreatitis (AP) on a cohort of 49 patients. Tethered bilayer lipid membranes (tBLMs) have been developed to investigate HSPs’ interactions with tBLMs that can be probed by electrochemical impedance spectroscopy (EIS). The results revealed that HSP70 and HSP90 interact via different mechanisms. HSP70 shows the damage of the membrane, while HSP90 increases the insulation properties of tBLM. These findings provide evidence that EIS offers a novel approach for the study of the changes in membrane integrity induced by HSPs proteins. Herein, we present an alternative electrochemical technique, without any immunoprobes, that allows for the monitoring of HSPs on nanoscaled tBLM arrangement in biologics samples such us human urine. This study demonstrates the great potential of tBLM to be used as a membrane based biosensor for novel, simple, and non-invasive label-free analytical system for the prediction of AP severity.

## 1. Introduction

Acute pancreatitis (AP) remains one of the most common gastrointestinal diseases. The most recent Modified Atlanta classification of AP includes three courses: mild (M), moderately severe (MS), and severe (S) [1]. M in AP progress is the most frequent (80%), and usually self-limiting, but around 20% go on to develop MS or S forms that involve pancreatic or peri-pancreatic tissue necrosis and/or organ failure. Mortality in those that develop sterile necrosis of the pancreas is reported to be 13% and, if there is infection, mortality can increase to up to 35% [2]. Obviously, markers associated with poor prognosis at early stages of hospital admissions might help to reduce the risk of death and severe complications due to AP. Many etiological factors are involved in the pathogenesis of AP, and its mechanisms should be studied.

**Figure 1:**
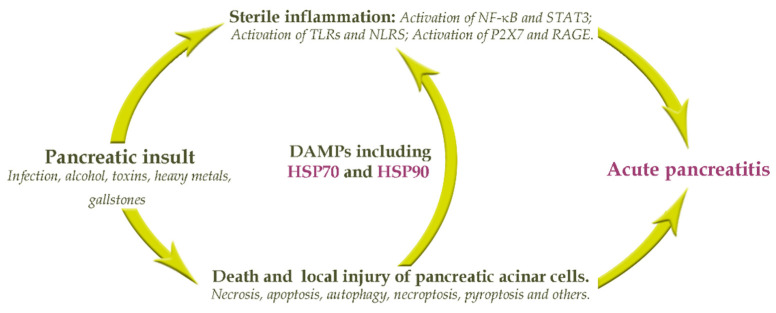
Principal scheme of cascade interactions in development of AP: Activation of nuclear factor-κB (NF-κB); mitogen-activated protein kinase (MAPK); signal transducer and activator of transcription 3 (STAT3); toll like receptor (TLR); nod-like receptors (NLRs); purinergic P2X7 receptor; receptor for Advanced Glycation Endproducts (RAGE); and damaged associated molecular patterns (DAMPs) [3].

The development of AP involves a complex cascade of pancreatic insult which has been described by Kang et al. [3]. On the basis of this study, the following principal scheme involving heat shock proteins (HSPs) can be presented (Figure 1). 

As shown in Figure 1, there are multiple processes initiated in the development of AP such as apoptosis, autophagy, necrosis, pyroptosis and others. When the injury or insult appears in the pancreatic acini and the protective barriers are damaged, multiple pancreas enzymes (such as lipolytic, proteolytic, amylolytic, and others) are leaked to the local tissues. This results in edema, vascular damage, hemorrhage, and cell death. Dead, dying, and injured pancreatic acinar cells release intracellular contents including damaged associated molecular patterns (DAMPs), which in turn promote the infiltration of various immune cells. Also, various inflammatory signaling pathways, including the activation of nuclear factor-κB (NF-κB), mitogen-activated protein kinase (MAPK), signal transducer and activator of transcription 3 (STAT3), toll like receptor (TLR), nod-like receptors (NLRs), purinergic P2X7 receptor, receptor for Advanced Glycation Endproducts (RAGE), and inflammasome and their associated key factors are activated. It appears that DAMPs, along with HSP70 and HSP90, play a central role in the pathogenesis of acute pancreatitis since DAMPs promote pancreatic tissue infiltration of many immune cells, leading to subsequent multiple organ failure and even death. Thus, the synergistic activation of HSP70 and HSP90 and their association with acute pancreatitis disease severity were investigated in our study. 

Previous studies showed that in the early stages of AP, local and systemic processes are activated, and digestive enzyme zymogens fuse with lysosomes where trypsinogen is activated to trypsin [4,5]. In addition, NF-κB dependent pro-inflammatory pathways are activated in the pancreas, leading to release inflammatory cytokines from acini [6]. The latter findings lead to injury at local and systemic levels during the acute phase of acute pancreatitis [4,6]. It has been shown that a number of pathways are involved in helping cells to survive stressful conditions. “Heat shock response” is one of these pathways [7]. In situations when cells undergo acute or chronic stress, such as AP, HSPs’ response is activated and the overexpression of these proteins then assists in restoring proper protein conformation and homeostasis. HSPs are mostly known as molecular chaperons to assist the folding and assembly of other proteins [8]. Horvath et.al. demonstrated the relationship of HSPs to pancreas inflammatory diseases (e.g., AP) [9]. Heat shock proteins, in particular HSP70 and HSP90, in acute pancreatitis have attracted more interest in the last decade. One of the most conserved and well-studied families of heat shock proteins is the HSP70 superfamily. According to Giri et al. these are ATP dependent chaperones that range in size from 66 to 78 kDa and are encoded by a set of 11 genes in humans [7]. The same study reports that the levels of HSP70 are transcriptionally regulated by “Heat Shock Factors” carrying tissue-specific pattern of expression [10]. Multiple studies described various methods such as thermal stress and others to overexpress HSP70 [7]. Another method wherein HSP70 played a crucial role in regulating inflammation response was the inhibition of the secretion of HMGB1. During the acute phase of acute pancreatitis, cell damage led to the release of cytokines, HMGB1 and HSP70 [11]. It was reported that HSP70 might play a protective role in AP since its decrease in concentration was associated with poor pancreatitis outcome, severity, and higher mortality. Another study by Lee et al. confirmed that an increase in the expression of HSP70 is associated with an improvement in survival, less tissue damage, and decreased inflammatory response [12].

Cytosolic HSPs may be released as a free form or can be bound to exosomes. Secreted HSPs may interact with other cells or be released into the bloodstream. Extracellular HSPs can mediate immunological functions and may act as potent danger signal, activating the immune system response. Additionally, it is a sign of many pathologies such as infection, stress, or cancer [13]. It was shown that HSP70 translocates into the plasma membrane after stress and alters the plasma membrane of acinar cells, allowing substances a size of 70,000 MW to enter the cytosol [14,15,16]. So far, while many techniques have been developed for detection of HSPs in various biological fluids, they are associated with several diseases, such as heart failure [17], seizure-related brain injury [18], or liver cancer [19] and can serve as markers for early clinical screening of depression [20]. Currently accepted methods for the detection of HSPs include enzyme-linked immunosorbent assay (ELISA) [17,18,19,21], immunofluorescence [22], Western blotting [23], qRT-PCR [24], and flow cytometry [21,22,24]. Although these methods have realized the sensitive determination of HSPs, the time-consuming and complex experimental process, derived from the peculiarities of an assessments of particular disease, makes the use of these techniques for wider applications limited. Moreover, most of these methods are based on immune recognition that is not only time-consuming, but also quite expensive and complicated. Also, several disadvantages to these approaches, including insufficient absorption, lack of consistency in laboratory accuracy, and interference from other molecules in the fluid environment, have been mentioned. 

It is likely that in recent years, with the rapid development of material science, many modified surfaces based on different conductive materials can signally enhance the application of electrochemical immunosensors and further achieve ultra-sensitive analysis [20]. Additionally, the optical biosensor based on the nanostructured porous silicon (PSi) with immobilized antibodies for label-free detection of HSP70 has been presented [25].

**Figure 2:**
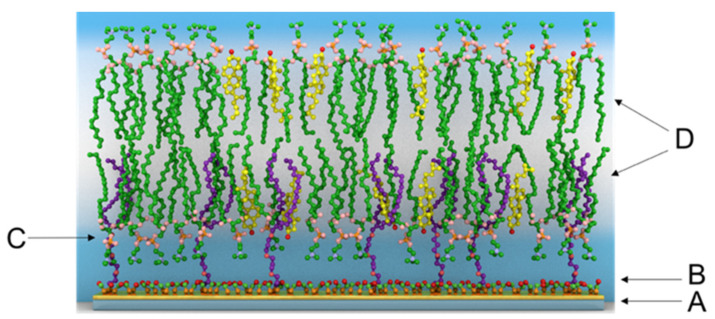
Illustration of the tBLM: **A**, Au-monolayer coated glass slide; **B**, β-mercaptoethanol; **C**, Anchor molecule; **D**, phospholipid bilayer. Mixed SAM defines an ionic reservoir between the gold and the bilayer membrane.

The modelling of potential mechanisms by interpretation of HSP interaction with membranes may help us to understand the pathogenesis of pancreatitis, as well as bring new clues for its clinical prevention and therapy [4]. Tethered bilayer lipid membranes (tBLMs) have been engineered on the Au-monolayer coated glass slide as a long-term stable and versatile experimental platform for protein reconstitution and for lipid–protein interaction studies (Figure 2) [10,26,27]. It has a nanoscale space between the solid substrate and bilayer for transmembrane protein insertion and free ion diffusion [28,29,30].

In this work, different isoforms of HSP’s were used to investigate their interaction with tBLM. Moreover, the tBLM damaging properties exhibited by selected proteins can be probed by electrochemical impedance spectroscopy (EIS). Due to the simplicity, low-cost, sensitivity, and rapid response, various electrochemical approaches are very promising nowadays. In our study, we have measured the serum concentrations of HSP70 and HSP90 in patients with AP by ELISA and we have analyzed the relationship of these to a degree of severity. Herein, we also describe an alternative simple, sensitive electrochemical technique which does not require any immunoprobes and allow for the monitoring the HSPs action on engineered tBLMs. Illustration of the tBLM molecular architecture is shown in Figure 2. EIS has been used extensively for the detection of biomolecules [31,32], and also for study of protein-lipid interactions [28,30,33].

We firmly believe that the use of tBLM and EIS offers a novel approach for studying the changes in the integrity of membrane arrangement induced by HSPs proteins, as well as for developing new non-invasive diagnostic tools.

## 2. Materials and Methods

### 2.1. Settings and Patient Data 

A total of forty acute pancreatitis patients’ samples of the same race and ethnicity and nine age/sex-matched healthy control samples were studied in accordance with the Institutional IRB approval (No. 158200-17-941-455). The ages ranged from 22 to 69 years old. The AP etiology was alcoholic in 19 (47.5%), biliary in 13 (32.5%) and idiopathic in 8 (20%). From the studied cohort, seven patients had lethal outcomes, peripheral blood and urine samples from the pancreatitis patients were collected on admission and following 3 consecutive days (24 h, 48 h, and 72 h since hospitalization). We conducted a prospective observational study and, all of the patients admitted to Vilnius University hospital “Santaros klinikos” with a diagnosis of acute pancreatitis and an onset of the diseases within the last 72 h were included in the study. The diagnosis was established on the basis of acute abdominal pain, with at least three-fold elevated levels of serum amylase and typical radiological findings. The contrast-enhanced computed tomographic (CT) scan was performed on days 5–7 after the onset of the disease. Clinical data related to the severity of disease as well as the development of organ dysfunction and/or septic complications were prospectively collected according to the clinical course and Modified Atlanta Classification. The patients were followed up with during their stay at the hospital until either the time for discharge or lethal outcome. The patients with underlying chronic pancreatitis and the patients with acute pancreatitis for more than 72 h were excluded from the study. HSP70 and HSP90 levels were measured using ELISA. All AP patients with no history of previous episode of the disease were admitted to the emergency room (ER) less than 24 h after the symptoms started. The diagnosis was confirmed by biochemical changes, clinical examination, and ultrasound examination. Disease severity (mild, moderate severe, severe) was evaluated using the Modified Atlanta Classification. If patients’ condition became unstable, they were treated at the intensive care unit (ICU).

### 2.2. Enzyme-Linked Immunosorbent Assay (ELISA)

Invitrogen™ HSP70 Human ELISA Kit and Invitrogen™ HSP90 alpha Human ELISA Kit (Thermo Fisher Scientific, Waltham, MA, USA) have been used for the quantitative detection of HSP70 and HSP90. 

### 2.3. Serum Sample Preparation

BD Vacutainer 3.5 mL SST(TM) II PET tubes with clot activator (silica) and separating gel were used for blood sampling. After clotting, blood samples were centrifuged at 2500× *g* for 10 min at room temperature. Serum aliquots were frozen immediately after centrifugation at (−70) °C or below. Prior to assay, the frozen samples were brought to room temperature slowly and mixed gently.

### 2.4. Principles of the HSP70/90 ELISA Test

An anti-human HSP70/90 coating antibody was adsorbed onto micro wells. Human HSP70/90 present in the sample or standard bound to antibodies adsorbed to the micro wells. After incubation, unbound sample components were removed during a wash step and a biotin-conjugated anti-human HSP70/90 antibody was added and bound to HSP70/90 captured by the first antibody. Following incubation, the unbound biotin-conjugated anti-human HSP70/90 antibody was removed during a wash step. Streptavidin-HRP was added and bound to the biotin-conjugated anti-human HSP70/90 antibody. After incubation, unbound Streptavidin-HRP was removed during a wash step, and substrate solution reactive with HRP was added to the wells. A coloured product was formed in proportion to the amount of human HSP70/90 present in the sample or standard. The reaction was terminated by the addition of acid and absorbance was measured at 450 nm. A standard curve was prepared from seven human HSP70/90 standard dilutions and the human HSP70/90 sample concentration was determined.

### 2.5. Urine Sample Preparations

Urine samples were collected from 12 healthy adult volunteers that had not shown symptoms of infection and had not taken antibiotics within 1 month prior to the sample collection. Samples were centrifuged at 1500× *g* for 10 min at room temperature, and the sediment of broken cells or tissues and other solid materials in the pellet were discarded. The urine samples were aliquoted and stored in a −80 °C freezer. Normal urine samples from healthy donors were supplemented with protease inhibitor cocktail (Sigma Aldrich, Hamburg, Germany) to avoid proteolysis. The pH of the resulting urine was measured to be pH 6.7. All collection and processing of the samples was performed and conformed to the Vilnius University ethical approval and clinical sample handling guidelines.

### 2.6. Tethered Phospholipid Bilayer Membrane (tBLM) Formation

Gold substrates for electrochemical impedance spectroscopy measurements were prepared on 25 by 75 mm glass slides (Thermo Fischer Scientific, Bremen, Germany). Gold layers were deposited by the magnetron sputtering using a PVD75 (Kurt J. Lesker Co., Jefferson Hills, PA, USA) system. For the formation of the self-assembled monolayer, the Au-coated glass slides were immediately immersed in ethanolic solutions of mixtures of HC18 and βME (in molar ratio 45:55) (c total = 0.2 mmol/L) and incubated at least 2.5 h for self assembly. The details on the synthesis and properties of the self-assembled monolayers used in this work were described earlier [26,27]. The arrangement of tBLM were completed by multilamellar vesicle fusion method described in previous papers [34]. A lipid vesicle solution was prepared from 1,2-dioleoyl-sn-glycero-3-phosphocholine (DOPC) and cholesterol (Avanti Polar Lipids, Alabaster, AL, USA) in molar % ratio 60:40 (for HSP70 interaction studies) and 75:25 (for HSP90 interaction studies) in a phosphate buffer solution containing 0.1 M NaCl (Roth, Denmark), 0.01 M NaH_2_PO_4_ (Roth, Frederikssund, Denmark), pH 4.5. Vesicles were rinsed with phosphate buffer solution (PBS) containing 130 mM NaCl, 2.7 mM, KCl 10 mM (Sigma Aldrich, Hamburg, Germany), Na_2_HPO_4_ (Sigma Aldrich, Hamburg, Germany), and 1.8 mM KH_2_PO_4_ (Sigma Aldrich, Hamburg, Germany), pH 7.4. 

### 2.7. Electrochemical Impedance Spectroscopy

EIS measurements were registered using the Zennium electrochemical workstation (Zahner GmbH, Kronach, Germany) between 0.1 Hz and 100 kHz, with 10 logarithmically distributed measurement points per decade. Data were fitted using ZView software (Scribner Associates, Southern Pines, NC, USA). All recording procedure and parameters are described in [26,27]. All measurements were carried out in sodium phosphate buffer solution (PBS) pH 7.4. A three-electrode cell configuration was used. A saturated silver-silver chloride (Ag/AgCl/NaCl (aq,sat)) microelectrode (M-401F, Microelectrodes, Bedford, NH) was used as a reference. The auxiliary electrode was a 0.25-mm-diameter platinum wire (99.99% purity, Aldrich) coiled around the barrel of the reference electrode. The working electrode was an Au-monolayer coated glass slide. EIS measurements in twelve separate electrochemical cells (volume, V ≈ 150 μL) on each slide, with the working surface area A_el_ ≈ 0.25 cm^2^ exposed to the solution. Measurements were carried out with 10 mV alternating current at 0 V bias versus the reference electrode in aerated solutions. All measurements were performed at room temperature (21 ± 2 °C). All of the values of the EIS parameters were normalized to a geometric surface area, A_el_.

### 2.8. Statistical Analysis

All experiments mentioned above were conducted three times or more. The statistical analysis and graphical display were performed using OriginPro 8.5. Comparison among groups was performed by one-way analysis of variance (ANOVA) followed by Tukey’s test. *p*-values less than 0.01 (*p* < 0.01) or 0.02 (*p* < 0.02) were regarded as statistically significant.

## 3. Results

### 3.1. HSP70 and HSP90 Assay in Serum

Aiming to determine the prognostic utility of serum HSPs for evaluation of severity level in the early assessment of AP clinical course, a cohort consisting of control (C), mild (M), moderately severe (MS), or severe (S) patients has been assessed. Among S patients, seven of them later had lethal outcomes (LO). The severity of AP was evaluated by clinical examination, laboratory (three times increase lipase/amylase levels) and radiological tests methodology and the concentrations of HSP70 and HSP90 in serum were detected by ELISA assessments. The OriginPro 8.5 was used to calculate an overall ANOVA Tukey test statistics for evaluation of both HSP90 and HSP70 concentrations found in serum vs. AP severity. 

**Figure 3:**
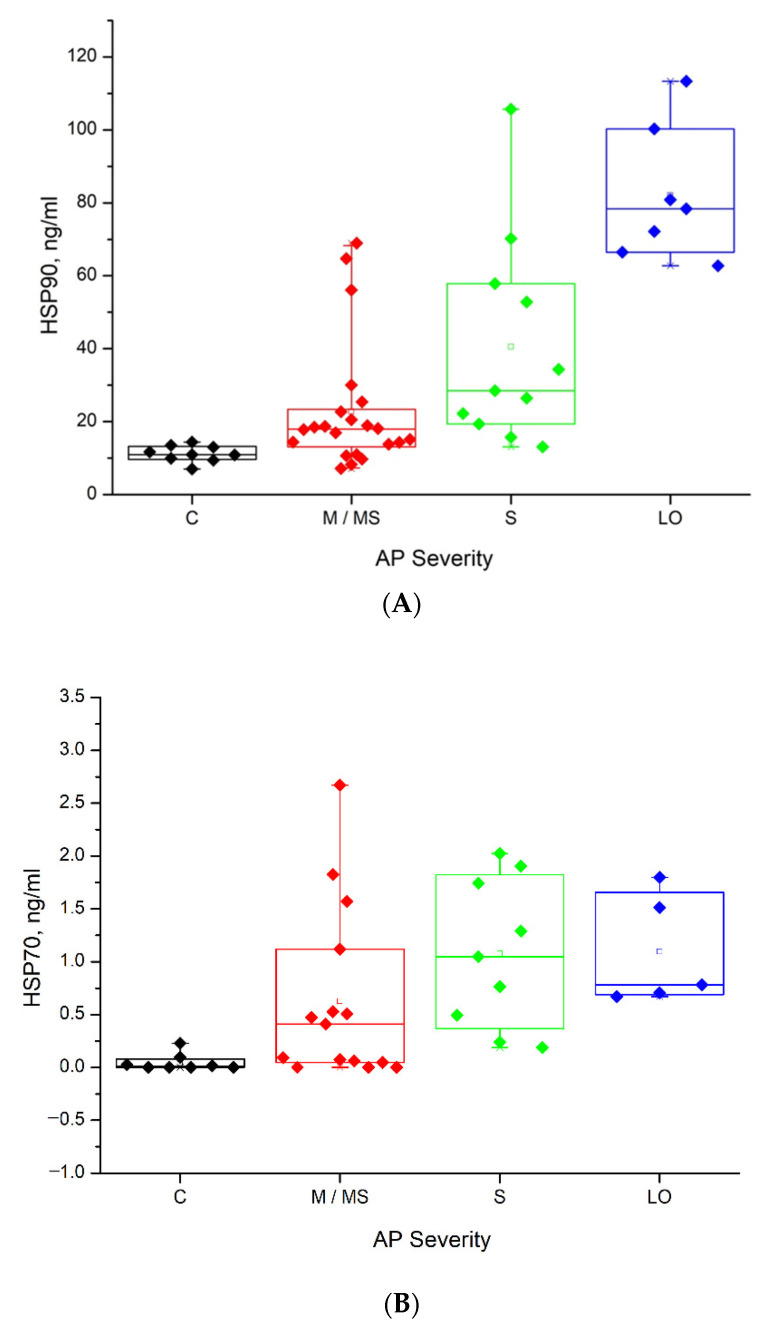
Concentrations of serum HSP90 vs. severity of AP: (**A**) *p* = 0.01 and HSP70; (**B**) *p* = 0.02.

The summarized data are shown in Table 1 and Figure 3A,B. 

As can be seen in Table 1 and Figure 3, the mean of the HSPs concentrations for both proteins are related to the course of AP. In the case of HSP90, it increased linearly with an increase in the severity of AP (Figure 3A). Overall ANOVA *p*-value was reported of 0.7 · 10^−9^. A *p*-value smaller than 0.01 means that a significantly different group was found. Patients with LO in AP had significantly higher concentrations of HSP90 compared to the C, M/MS, and S AP groups. Patients with the S level of AP also had significantly higher concentrations of HSP90 comparing to C group. 

However, in the case with HSP70, the relationship was not linear. In the groups from C to M/MS the mean of the concentration rose from 0.05 ng/mL to 0.63 ng/mL, reaching a maximum of 1.08 ng/mL in the group S. In the LO group, it decreased (Table 1, Figure 3B).

In the case of HSP70, the overall ANOVA *p*-value was reported to be 0.012. This *p*-value, smaller than 0.02, indicates that patients with S in AP were found to have significantly higher concentrations of HSP70 than those in the control group. However, no statistically significant differences were found between groups of S and M/MS of AP patients. In contrast to the case of HSP90, the LO group in HSP70 assessments possessed a lower mean of HSP70 concentration compared to the S group. 

### 3.2. Interaction of HSPs with Phospholipid Model Membranes by EIS 

**Figure 4:**
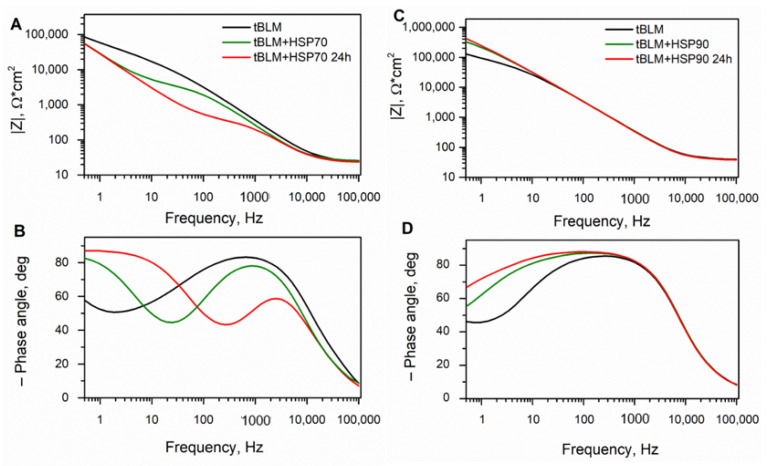
Interaction between HSP70 and HSP90 with tBLM: (**A**) impedance magnitude vs. frequency (Bode) plots and (**B**) Phase angle plot of EIS spectra: black, the initial spectra of tBLM; green, 10 min after HSP70 of 0.2 μM injected; red, 24 h after injection of HSP70. tBLM was composed from DOPC:CHO (in ratio % 60:40); (**C**) Impedance magnitude vs. frequency (Bode) plots; and (**D**) Phase angle plot of EIS spectra: black, the initial spectra of tBLM; green, 10 min after HSP90 of 2 μM injected; red, 24 h after injection of HSP90. tBLM was composed from DOPC and Cholesterol (in ratio % 75:25).

Since the mechanism of HSP70 and HSP90 acting during AP is largely unknown, we proposed to investigate an interaction by reconstitution of these proteins into model systems (i.e., tethered bilayer lipid membranes). The effect of reconstitution of HSP70 and HSP90 into the tBLM is shown in Figure 4. HSPs’ action is readily seen from the changes in the EIS plots. In particular, upon incubation of tBLMs with HSP70, a step-like kink develops on a |Z| vs. frequency (Figure 4A). In addition, the phase minimum appears, and it moves towards higher frequencies as the incubation time of HSP70 increases (Figure 4B). Phase shift of EIS spectra after interaction with HSP70 shows the damage of the membrane and alters the ion movement through the membrane (Figure 4A,B). Similarly, EIS spectral signatures have been reported after membrane incubation with pore-forming toxins [29,35,36], all of which increase the conductivity of membranes. The interaction of HSP90 with tBLM, composed from DOPC:CHO (in ratio % 75:25), is shown in Figure 4C,D. Such tBLM composition has been selected based on the literature and on EIS data (Figure S1 and Table S1). EIS data displayed a stronger interaction of HSP90 on the 25% cholesterol-containing membranes. The opposite result with the interaction of HSP70 was observed. The introduction of HSP90 to tBLM increases |Z|, in the low frequency range (Figure 4C), and triggers a shift of the phase minimum towards lower frequencies as the incubation time of HSP90 increases (Figure 4D).

Such a phase shift after interaction with HSP90 indicates that proteins are attaching to the membrane and blocking any ion movement through the membrane. These findings are in agreement with the previous studies by Zhang M. et al., which showed a strong association followed by weak dissociation of HSP90 with lipid membrane [37]. To quantify the effect of HSPs on the electrical parameters of the tBLMs, |Z| was measured at the frequency point, f_min_, at which the minimum of the negative of impedance phase is observed [38,39]. Since the extent of membrane damage and defectiveness could be more conveniently related to the conductance of tBLMs at f_min_ (estimated as Y = 1/Zphase min) is used to characterize the interaction between HSPs and the tBLMs. 

Table 2 summarizes the observed tBLM conductance data from the EIS spectra (Figure 4). The mean values of conductance for the prepared tBLM in absence of HSPs was Y = 20.2 μS/cm^2^, determined from *n* = 20 independent measurements. The damage of tBLM inflicted by HSP70 is clearly demonstrated by the data in Table 2. An increase of more than ten times of the conductance of tBLM with value 275.7 μS/cm^2^ was detected after incubation with HSP70, clearly indicating that the integrity of the membrane was affected by HSP70. In contrast, after incubation with HSP90, a decrease of conductance of the tBLM was observed. 

These findings (Figure 4 and Table 2) indicate that EIS spectra changes generated by HSP70 possess different characteristics compared to the tBLM conductances generated by the presence of HSP90. Obviously, the tBLM interaction with HSP70 exhibits the damaging properties of the membrane. Meanwhile, the binding observed between HSP90 and tBLM significantly decreased the conductance and capacitance of tBLM (Table 2). 

### 3.3. HSP70 Action on Tethered Lipid Membranes in Urine Samples

**Figure 5:**
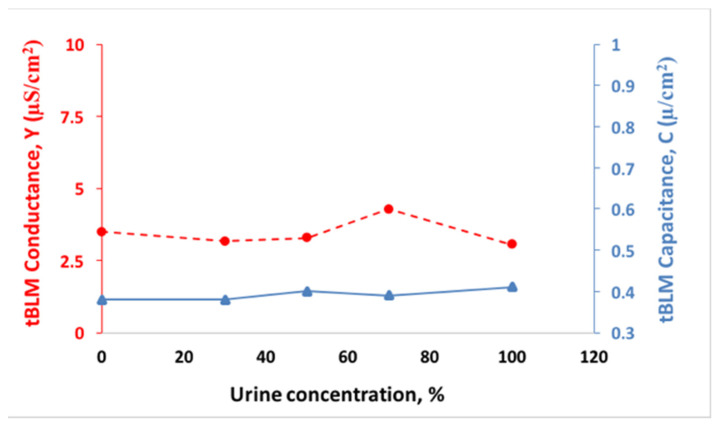
The effect of urine on tBLM capacitance (right axis, triangles/solid line) and conductance (left axis, circles/dashed line) composed from DOPC:CHO (in ratio % 60:40)) as a function of urine concentration. The urine impact on tBLM conductance (circles/dashed line) and on tBLM capacitance (squares/solid line) is shown.

In order to demonstrate the applicability of the tBLMs for the sensing of HSPs in real biological media of acute pancreatitis patients, we started with the detection of HSP70 in urine of healthy adults. EIS allowed to evaluate the possible specific or non-specific interactions of urine components with the sensor (i.e., the arrangement of tethered lipid bilayer on Au-coated monolayer). The conductance, Y, and membrane capacitance, C, of tBLMs completed with DOPC:CHO (in ratio % 60:40) are plotted in Figure 5 as a function of urine concentration. The conductance and capacitance of tBLM remained constant and were not affected by urine components (Figure 5). A minimal non-specific adsorption of urine components on tBLM was observed, which did not affect the sensitivity of the EIS response. The EIS spectra of these data are shown in Figure S2.

**Figure 6:**
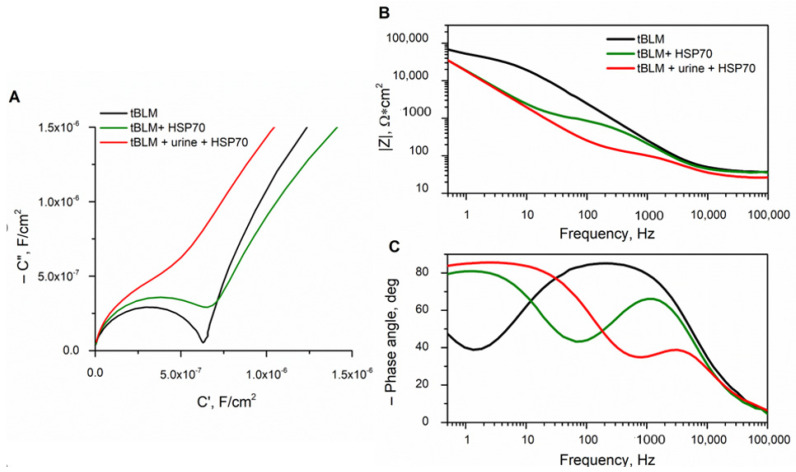
Comparison of the impact of HSP70 on tBLM in urine (red) and buffer (green) media: (**A**) The Cole–Cole plot of complex capacitance. Spectra is enlarged high-frequency part of the spectrum. The curves refer to the same Bode spectra as in B and C; (**B**) impedance magnitude vs. frequency (Bode) plots; and (**C**) impedance phase vs. frequency (Bode) plots. tBLMs were completed with the DOPC:CHO (in ratio % 60:40) phospholipid mixture. HSP concentration was kept 0.05 µM. The sample size for this experiment was *n* = 12.

In this study, amounts of HSP70 were added into the buffer and in urine to obtain different concentrations of HSP70 in these media. The sensitivity of tBLM for HSP70 detection was investigated in this study by comparing the EIS response in buffer and in urine samples. The addition of HSP70 into the buffer and urine solutions in contact with tBLMs induced changes in the EIS spectra (Figure 6A–C). The enlarged high-frequency part of the complex capacitance plots of the EIS spectra as a function of the HSP7O concentration in buffer and urine are shown in Figure 6A. The full Cole–Cole plot exhibits a typical “two semi-circle” shape, which is a characteristic of disrupted integrity of tBLM (Figure S3). From the mathematical analysis by Valincius et.al, such changes in the Cole–Cole EIS plots signals the increase in the membrane defect density (Figure 6A) [38,40]. The greater the damage of the tBLM, the higher the frequency edge at which the onset of the capacitive behavior was observed. The presence of HSP70 leads also to a characteristic reduction of the membrane impedance on the Bode plots (Figure 6B). In both cases, EIS spectra plateau appears, which drops down after interaction with HSP70, signalling an increase of the conductance of tBLMs (Figure 6B). Additionally, on a plots phase vs. frequency a minima develops, which shift towards high frequency edge after interaction with HSP70 (Figure 6C). All summarized and calculated tBLM parameters from EIS spectra (Figure 6A–C) are presented in Table S2. All of these features of EIS spectra exhibit that the HSP70 action on tBLM is stronger in urinary medium compare to a buffered medium.

**Figure 7:**
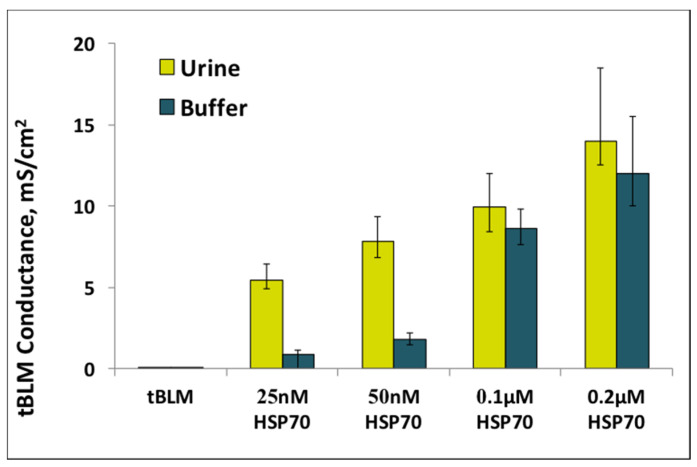
The effect of HSP70 on the conductance of BLMs, composed from DOPC: CHO (in ratio % 60:40) phospholipid mixture, as a function of protein concentration in urine (yellow) and buffer (teal) in range from 25 nM to 0.2 µM. The spectra were measured 10 min after the addition of HSP70 to final concentration.

Figure 7 shows a summarized histogram and Table S3 of tBLM response to HSP70 in buffer and in urine media. The HSP70 induced tBLM conductance (Y_tBLM_) as a function of HSP70 concentration in urine and in buffer media is presented. Value of Y_tBLM_ was increased gradually in not linear function upon increasing concentrations of HSP70. 

The increase of conductance of tBLM indicates the damage of membrane by HSP70. Data presented in Figure 7 indicates a significant, almost five-fold increase in the conductance of tBLM for the HSP70–tBLM interaction in urine as compared to that in buffer, at low HSP70 concentrations from 25 nM and 50 nM. Such sensitizing properties of urine might be useful in practical applications and opens new possibilities for applying such systems in non-invasive diagnostics.

## 4. Discussion

This study clearly showed that the heat shock proteins HSP90 and HSP70 are expressed during the development of acute pancreatitis (AP) and influence the course of AP. It was explored on a base of a wide cohort consisting of 49 patients. The established dependence of HSPs on the severity level of AP is very important since it can be associated with poor prognosis at early stages of hospital admissions and might help to reduce a risk of death and severe complications in AP. It was found that the mean of HSP90 concentrations in serum increased linearly by increasing the severity of AP (Figure 3A). In the case of HSP70, the relationship was not linear. The LO group in HSP70 assessments possessed a lower mean of HSP70 concentration compared to the S group (Figure 3B). The authors of study [11], on the basis of two lethal outcomes, demonstrated that low HSP70 was associated with poor prognosis and concluded that HSP70 might play a protective role in AP. A decreasing tendency of the HSP70 amount for the LO group obtained in this study supports this finding. The proposition that the heat shock response in AP activated via HSP70 protects acinar cells was also demonstrated in [7]. It inhibits trypsinogen activation and modulates NF-κB signalling to limit acinar cell injury. However, evidently lower concentrations of HSP70 for the M and control groups (compared to LO) suggest that some other aspects can influence the protective role of HSP70 in the illness. In addition to other already known aspects [41], the results of this study revealed the importance of HSP90, since the amount of HSP90 in contrast to HSP70 was highest in the LO group consisting of seven patients, including the same patients as in a case with HSP70. This study gave strong experimental evidence that both HSPs are expressed during the development of AP and, also, that both proteins influenced the course of AP. On the other hand, investigations concerning the dependences of certain amounts of HSP70 and HSP90 vs. AP severity revealed that their influence to the illness is different. Hence, HSP70 plays a protective role and HSP90, supposedly, complicates the recovery process [11]. From an analytical point, the determination of HSPs’ concentration is important since it can be used for the prediction of the progression of AP. The data presented in Table 1 show that in the case of HSP90, the highest concentration of 66.5–100 ng/mL is associated with lethal outcomes while the concentration range within 19.0–57.7 ng/mL is associated with the severe course of AP. In the case of HSP70, the S or lethal outcomes are associated with a concentration of 0.36–1.82 ng/mL. 

The physical mechanism of the interaction between the HSP70, HSP90, and cell membrane is not clear. Previous studies had shown a strong interaction of HSP70 [42] and HSP90 [37] with phospholipid membranes. The pore-forming mechanism by HSP70 was also presented by Arispe [43], where HSP70 channel activity showed stable trains of brief current spikes, indicating brief multiple channel conductance changes. Also, the SPR sensorgrams for the interaction of HSP90 with membrane lipids showed a strong binding of HSP90 [37]. This research was conducted in order to monitor the HSPs’ action on model membranes and to assess the possible mechanisms of membrane damage.

In this study, the tethered lipid bilayer membranes (tBLMs) on Au-coated monolayers were engineered, optimized and applied as a valuable experimental model platform for label-free detection of different characteristic interactions demonstrated by HSPs. It has been examined that HSP70 and HSP90 interact with the tBLM via different mechanisms. HSP70 induces a conductance of the membrane (Figure 4A,B), while HSP90 increases the insulation properties of tBLM (Figure 4C,D). The results are in agreement with those presented in Figure 3, where it was confirmed that both HSPs are associated with the severity of AP and are likely to affect the lethal outcomes in AP differently. 

Assessments of HSPs performed in serum revealed that they can serve as the pathogenic biomarkers associated with AP severity (Figure 3). Bearing in mind that urine samples are non-invasive and easy to collect, HSPs’ analysis in urine would be of great value for wider diagnostic monitoring. The proposed method is prospective since damage by HSP70 inflicted on the dielectric properties of tBLMs can be detected by electrochemical impedance spectroscopy (EIS) and do not require any immunoprobes. The use of tBLM made it possible to develop a new method for the detection of HSP70, which would allow for analysing HSP70 in urine. The minimal non-specific adsorption of urine components was detected, which did not affect the sensitivity of the sensor (Figure 5). These findings allowed us to further apply the tBLM platform for the determination of HSPs in non-invasive biological fluid, namely urine. A significant, almost five-fold increase in the conductance of tBLM for the HSP70–tBLM interaction in urine, as compared to that in buffer, was obtained (Figure 6). Such sensitizing properties of urine might be useful in practical applications where the sensitivity of the tBLMs system is crucial for the detection of small amounts of membrane damaging HSPs. Alternatively, direct measurements of changes in membrane permeability have been reported by Perini et.al. [44,45]. In such studies, cells were labelled with laurdan (2-dimethylamino-6-lauroylnaphthalene), a polar sensitive fluorescent membrane probe for measuring changes in membrane permeability. Laurdan is widely used to study lateral organization of membranes in either artificial or natural systems. In future studies, we will consider to use this probe in membrane permeability studies, which could certainly provide a useful information on the structure of the membranes and also could give more information on the action of HSP70 on lipid membranes.

In conclusion, the presented analysis provides a practical tool to evaluate the conductivity of tBLMs which could be utilized in tBLM based biosensor applications. Currently the sensitivity of our tBLM based sensor for detecting HSP70 is close to 25 nM (≈1750 ng/mL). This concentration is similar with that achieved by novel optical biosensor based on the immunoassay, where a limit of detection of 1290 ng/mL was observed [25]. Although the sensitivity achieved in this work does not reach that of current ELISA methodologies and electrochemical immunosensors, it is capable of detecting HSP70 down to ≈0.02 ng/mL [20]. However, it should be noted that the sensitivities presented above are achieved not in urine. Therefore, on the basis of these studies, it can be expected that the tBLM is a valuable experimental label-free platform for the detection of HSP70 in urine. It opens up new possibilities for the application of such systems as potential markers for early prognostics or non-invasive diagnostics in the development of AP. Since the current sensitivity of proposed non-invasive methods is not sufficient enough, a higher sensitivity will become the focus of our subsequent research.

## Figures and Tables

**Table 1:** The statistical data of HSPs concentration found in control (C), mild (M), moderately severe (MS), severe (S), and lethal outcomes (LO) groups.

	HSP70		HSP90	
	Mean, ng/mL	25th and 75th percentiles range, ng/mL	Mean, ng/mL	25th and 75th percentiles range, ng/mL
C	0.05	0.007–0.09	11.2	9.6–13.2
M/MS	0.63	0.05–1.12	22.8	13.0–23.1
S	1.08	0.36–1.82	40.6	19.0–57.7
LO	1.09	0.68–1.65	82.0	66.5–100

**Table 2:** Conductance of tBLM before and after incubation with HSP70 and HSP90. tBLM was composed from DOPC:CHO (in ratio % 60:40) in case of HSP70 and (in ratio % 75:25) in case of HSP90.

	tBLM Conductance, Y (µS/cm^2^)	
	HSP70	HSP90
10 min	275.7 ± 54.3	3.4 ± 1.7
24 h	2584 ± 315	2.5 ± 0.7
tBLM	20.2 ± 13.1	

## Data Availability

The data set supporting the conclusions of this article is included within the article. Raw image data is available upon request to the corresponding author.

## Supplementary Materials

The following are available online at https://www.mdpi.com/article/10.3390/biomedicines9070755/s1: Figure S1, The comparison of EIS spectra of interaction between HSP90 and tBLM containing 40% of cholesterol and 25% of cholesterol; Table S1, The conductance of tBLM of the EIS spectra (Figure S1) of tBLMs containing 40% and 25% of cholesterol before and after interaction after HSP90; Figure S2, EIS spectra of tBLM completed with DOPC and Cholesterol (in ratio % 60:40) as a function of urine concentration; Figure S3, The complex capacitance plot of EIS spectra. Comparison of the impact of HSP70 on tBLM in urine (red) and buffer (green); Table S2, The calculated conductance and capacitance of tBLM after incubation with HSP70 in buffer in urine media; Table S3, The calculated conductance and capacitance of tBLM as a function of HSP70 concentration in urine and buffer in range from 25 nM to 0.2 µM.
