# Supplementary material for: Tethered Lipid Membranes as a Nanoscale Arrangement towards Non-Invasive Analysis of Acute Pancreatitis
Source: Biomedicines. 2021 Jun 29;9(7):755. doi: 10.3390/biomedicines9070755 (PMC8301313; doi:10.3390/biomedicines9070755)
Supplement: Supplementary file 1 [file biomedicines-09-00755-s001.zip › biomedicines-1264353-supplementary.pdf]

Supporting Information

Tethered lipid membranes as a nanoscale arrangement towards non-invasive analysis of Acute Pancreatitis

Rima Budvytyte[*]*, Akvile Milasiute, Dalius Vitkus, Kestutis Strupas, Aiste Gulla, Ieva Sakinyte, Julija Razumiene**

Experimental section

Materials

Recombinant Protein HSP70 was purchased from Sigma Aldrich (Germany) with purity of ≥70%. Recombinant HSP90 full length was donated by prof. D. Matulis from VU Life Sciences Center, Institute of Biotechnology, HSP90 was cloned and purified as previously described [1]. Lipids 1,2-dioleoyl-sn-glycero-3-phosphocholine (DOPC), and cholesterol were purchased from Avanti Polar Lipids, Inc. (Alabaster, USA) and used as received. All other solvents (AR grade) were used without purification.

Interaction of HSPs with tethered lipid bilayer membranes (tBLM) by EIS

Interaction of HSP90 with phospholipid model membranes composed from DOPC : CHO (in ratio % 60 : 40) and DOPC : CHO (in ratio % 75 : 25). For further studies, tBLM composition has been selected based on the literature[2] and on EIS data (Figure S1 A, B and Table S1). There were no changes in EIS spectra observed after HSP90 incubation with tBLM, containing 40% of cholesterol (Figure S1 A) Clearly, the stronger interaction of HSP90 on a 25 % cholesterol-containing membranes was indicated. (Figure S1 B).

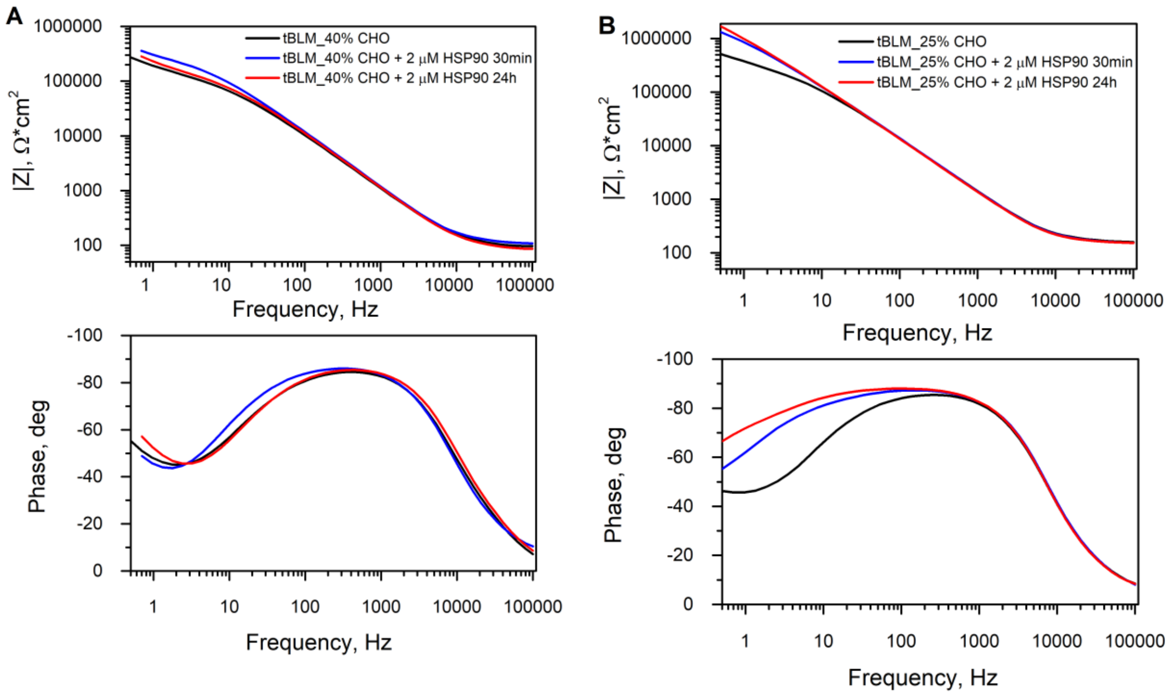

Figure S1. The comparison of EIS spectra of interaction between HSP90 and tBLM containing 40% of cholesterol and 25% of cholesterol. A - Phase angle vs frequency plot of EIS spectra. tBLM was composed from DOPC and Cholesterol (in ratio % 60 : 40). B – Phase angle vs frequency plot of EIS spectra. tBLM was composed from DOPC and Cholesterol (in ratio % 75: 25). Black – the initial spectra of tBLM, blue – 30 min after HSP90 of 2 μM injected, red – 24 hours after injection of HSP90.

Table S1 summarizes the calculated conductance of tBLM from EIS spectra (Figure S1) after interaction of HSP90 with tBLM, containing 40% and 25% of cholesterol. The more pronounced binding of HSP90 were obtained in membranes containing 25% of cholesterol. In this case the conductance of tBLM decreased 4 times after interaction with HSP90. Meanwile during the interaction of HSP90 and tBLM containing 40% of cholesterol the conductance of tBLM remained unchanged.

Table S1. The conductance of tBLM of the EIS spectra (FigureS1) of tBLMs containing 40% and 25% of cholesterol before and after interaction after HSP90.

	tBLM conductance, Y (μS/cm^2)
	**DOPC and Cholesterol (60 : 40)**
tBLM	26.4
30 min	16,64
24 val	26
	**DOPC and Cholesterol (75 : 25)**
tBLM	10,4
30 min	3
24val	2,48

HSP70 Action on Tethered Lipid Membranes in Urine Samples

Minimal non-specific adsorption of urine components on tBLM was observed, which did not affect the sensitivity of the EIS response (Figure S2 A, B). Both Cole-Cole plot of complex capacitance and phase angle vs frequency plots of EIS spectra did not changed. It shows that electrical properties of tBLM were not affected by urine components.

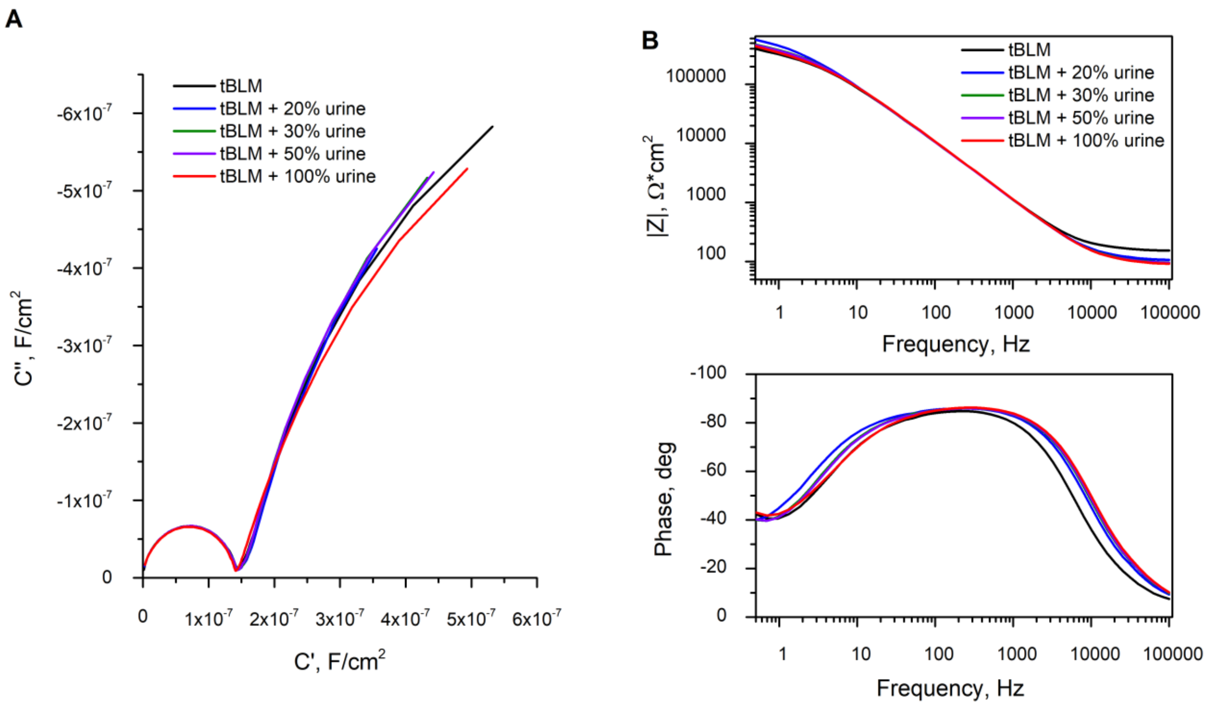

Figure S2. EIS spectra of tBLM completed with DOPC and Cholesterol (in ratio % 60:40) as a function of urine concentration. A – Cole-Cole plot of complex capacitance of EIS spectra. B - Phase angle vs frequency plot of EIS spectra. No urine (black); 20 % urine (blue); 30 % urine (green); 50 % urine (maroon); 100 % urine (red).

Addition of HSP70 into the buffer and urine solutions in contact with tBLMs induced changes in the EIS spectra. The full complex capacitance plots of the EIS spectra as a function of the HSP7O concentration in buffer and urine are shown in Figure S3. The Cole-Cole plot in full range of frequency from 0,5 Hz till 100000 Hz, exhibit a typical "two semicircle" shape, which is a characteristic of disrupted integrity of tBLM[3] (Figure S3).

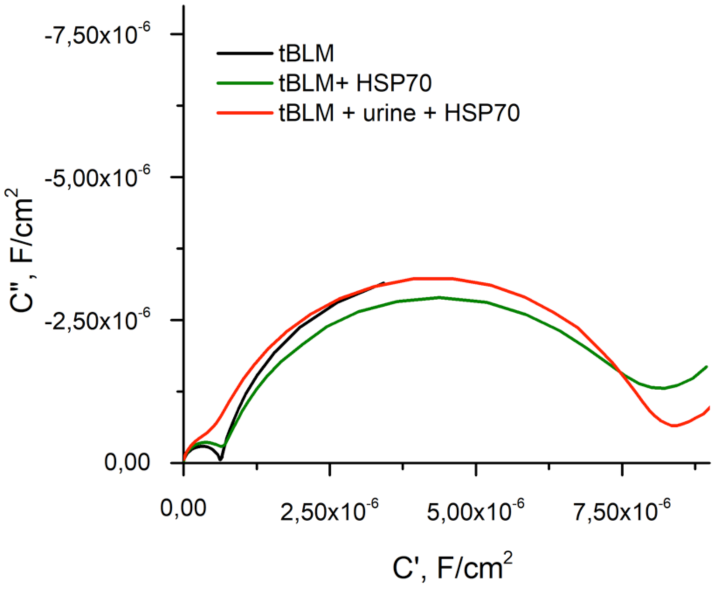

Figure S3. The complex capacitance plot of EIS spectra**.** Comparison of the impact of HSP70 on tBLM in urine (red) and buffer (green). A). The full range of frequency was from 0,5 Hz till 100000Hz. tBLMs were completed with the DOPC/CHO (in ratio % 60 : 40) phospholipid mixture. HSPs concentration was kept 0.05 μM.

The calculate conductance and capacitance of tBLM from EIS Spectra (Figure 5) after incubation with HSP70 in buffer and urine media is shown in Table S2. After interaction with HSP70 in buffer media, the conductance of tBLM has reached 1072 μS/cm^2, while in urine media it was 8 times bigger with value of 8660 μS/cm^2. The decrease of tBLM capacitance was observed after interaction with HSP70 in urine media, indicating non- specific interaction of urine components. Clearly, results concluding the action of HSP70 on membranes being stronger in urine media.

Table S2. The calculate conductance and capacitance of tBLM after incubation with HSP70 in buffer ir urine media. tBLM was composed from DOPC and Cholesterol (in ratio % 60:40) in case of HSP70. HSPs concentration was kept 0.05 μM.

	tBLM Conductance, Y (μS/cm^2)	tBLM Capacitance, C (μF/cm^2)
tBLM + HSP70	1072 ± 533	0.65 ± 0,03
tBLM + 100% urine + HSP70	8660 ± 1567	0.39 ± 0.05
tBLM	26.6 ± 5.7	0.64 ± 0.02

A summarized Table S3 of calculated conductance and capacitance of tBLM as a function of HSP70 concentration in urine and buffer media. The concentration range of HSP70 was from 25 nM to 0.2 μM. In both cases, the conductance of tBLM has increased gradually in not linear function upon increasing concentrations of HSP70.

Table S3. The calculated conductance and capacitance of tBLM as a function of HSP70 concentration in urine and buffer in range from 25 nM to 0.2 μM. tBLM was composed from DOPC and Cholesterol (in ratio % 60:40).

	tBLM conductance, Y (μS/cm^2)	tBLM Capacitance, C (μF/cm^2)
	**Buffer**	
tBLM + 0.025 uM HSP70	562 ± 123	0.65 ± 0,03
tBLM + 0.05 uM HSP70	1072 ± 533	0.36 ± 0.07
tBLM + 0.2 uM HSP70	10204 ± 5679	0.2 ± 0.05
	**Urine**	
tBLM + 0.025 uM HSP70	4365 ± 554	0.60 ± 0,04
tBLM + 0.05 uM HSP70	8660 ± 1567	0.39 ± 0.05
tBLM + 0.2 uM HSP70	16156 ± 4340	0.25 ± 0.06
tBLM	16.8 ± 5.7	0.64 ± 0.02

References

1. Cikotiene I, Kazlauskas E, Matuliene J, Michailoviene V, Torresan J, Jachno J, Matulis, D. 5-aryl-4-(5-substituted-2,4-dihydroxyphenyl)-1,2,3-thiadiazoles as Inhibitors of HSP90 Chaperone. *Bioorg. Med. Chem. Lett.* 2009;**19(4):**1089–1092.DOI: 10.1016/j.bmcl.2009.01.003

2. Zhang M, Wang D, Li P, Su C, Rong X, Gen Z, et.al. Interaction of Hsp90 with Phospholipid Model Membranes. *BBA–Biomembranes,* 2018;**1860(2):** 611–616. DOI: .org/10.1016/j.bbamem.2017.11.011.

3. Valincius G, Meškauskas T, Ivanauskas F. Electrochemical Impedance Spectroscopy of Tethered Bilayer Membrane. *Langmuir,* 2012;**28(1):**977-990. DOI: 10.1021/la204054g.
